# Urgent but isolated: Public perception and understanding of One Health in daily life

**DOI:** 10.1016/j.onehlt.2026.101623

**Published:** 2026-10-16

**Authors:** P. Saria, E.-M. Bartl, S. Winkler, R. Liermann, D. Ernst, S. Kau-Strebinger

**Affiliations:** a Centre for Food Science and Veterinary Public Health, Clinical Department for Farm Animals and Food System Transformation, University of Veterinary Medicine Vienna, Austria; b Clinical Centre for Ruminants, Camelids and Herd Health Management, Clinical Department for Farm Animals and Food System Transformation, University of Veterinary Medicine Vienna, Austria; c Messerli Research Institute of Human Animal Interactions, Department of Interdisciplinary Life Sciences, University of Veterinary Medicine Vienna, Austria; d Clinical Centre for Population Medicine in Fish, Pig and Poultry, Clinical Department for Farm Animals and Food System Transformation, University of Veterinary Medicine Vienna, Austria; e Centre for Pathobiology, Department of Biological Sciences and Pathobiology, University of Veterinary Medicine Vienna, Austria; f One Health PhD Programme, University of Veterinary Medicine Vienna, Austria

**Keywords:** One Health, Public understanding, Public engagement, Systems thinking, Food consumption, Animal husbandry, Environmental health, Health communication

## Abstract

Effective One Health (OH) implementation relies heavily on public cooperation, yet how the public perceives daily life OH intersections remains understudied. We investigated this baseline understanding via a targeted survey conducted during an open house event at the University of Veterinary Medicine Vienna (*n* *=* 92 responses). When presented with an everyday scenario–how preparing a fresh salad might inadvertently link global health, animal husbandry, and the environment–most participants cautiously acknowledged a connection, though only 10.9% accurately identified the underlying systemic linkages, suggesting a tendency toward overgeneralization. While recognised urgency for integrated veterinary, human, and environmental health solutions was high (83.7% rating OH action as highly urgent), open-ended mapping of everyday OH encounters predominantly focused on isolated categories like “Environment & Pollution”, “Everyday Life”, and “Food & Consumption”; leaving animal and agricultural dimensions critically underrepresented. These findings reveal a striking paradox of strong public motivation alongside fragmented OH understanding. We conclude that bridging this gap requires targeted public education utilizing brief, tangible daily examples to translate high systemic anxiety into actionable literacy. Limitations include priming and a modest, non-representative sample.

## Highlights

• Perceived One Health urgency conflicts fragmented literacy among the public.
• Overgeneralisation of health linkages obscures nuanced daily health risk perception.
• Public One Health mapping centers on environment, pollution, and food.
• Animal husbandry and agriculture are undervalued in an everyday One Health context.
• We need practical, well-rounded education turning concern into true health literacy.

## 1 Introduction

The One Health (OH) concept arose during the 20th century as an integrated, unifying and interdisciplinary strategy that recognises the interdependence of human, animal and environmental health [1], [2], [3]. It highlights that complex contemporary health challenges, including antimicrobial resistance, emerging and infectious diseases, food safety and vector borne diseases, cannot be effectively addressed within traditional disciplinary boundaries, requiring cross-sector collaboration instead [4], [5].

In recent years, the OH concept has gained traction within academic, policy and international health communities, driven by global events greatly impacting public health, including emerging zoonotic outbreaks and the COVID-19 pandemic [6]. However, despite its growing institutional recognition, public awareness and understanding of the OH framework remain comparatively limited [7]. Fostering public recognition is important, as societal engagement plays a critical role in supporting interdisciplinary health strategies and promoting behavioural and policy changes necessary to address interconnected global health challenges [8]. This interdependence is further highlighted by the fact that many Sustainable Development Goals are advanced by OH initiatives, including those focusing on good health (SDG 3), food security (SDG 2), clean water (SDG 6), climate action (SDG 13), and biodiversity (SDGs 14–15) [9]. While there are initiatives to integrate the OH concept into public education in Austria [10], little is known about the baseline understanding of this concept among the general public [11]. Consequently we conducted a targeted survey during an open house event at the University of Veterinary Medicine Vienna to evaluate public perceptions of everyday OH intersections.

## 2 Materials and methods

The OH PhD Programme at the University of Veterinary Medicine Vienna hosted an information booth as a third-mission activity during an open house day (Oct. 8th 2025) that drew nearly 5000 visitors. The main audience were prospective students, families with children, especially pupils as well as former students of the University and people generally interested in science and animals. Visitors were asked to fill out a questionnaire about OH when visiting the booth.

The survey was conducted anonymously and voluntarily. No personal data or identifying information were collected, and the results are reported only in aggregated form. Therefore, no personal data within the meaning of Art. 4(1) GDPR were processed; see also Recital 26 GDPR. Participants were provided age-appropriate information regarding the study's purpose. To verify informed consent, only questionnaires with a completed “consent and confirmation of the data protection declaration” checkbox were included in the evaluation. Respondents were given the option to complete the survey physically or digitally via a QR code.

The first part of the questionnaire summed up participants' profile, collecting data on age, gender and education and/or occupation. The detailed item topics are summarised in Table 1. The full survey in German, as well as the translated English version is provided in the Supplementary Fig. 1. Question 1 (Q1) assessed participants' estimated likelihood of an everyday, seemingly harmless activity–preparing a fresh salad–being linked to a broader global health issue. The question was designed to contrast a familiar daily routine with the latent, systemic risk inherent to OH challenges. Q2 tested the knowledge of various OH topics by asking participants to identify a specific cause for a health impact linked to eating and preparing salad. To highlight potential knowledge gaps and misconceptions within the sample, all provided response options were constructed to hold high narrative plausibility; however, only one option holds scientifically true by correctly linking animal husbandry, the environment, and human health. Q3 evaluated participants' perceived urgency regarding systemic solutions. This item was designed to gauge public endorsement of the core OH framework by measuring how strongly respondents value transdisciplinary collaboration across veterinary medicine, human medicine, and environmental sciences. The open-ended Q4 aimed to encourage people to reflect on personal OH experiences by providing concrete daily life examples. This question allowed respondents to freely identify real-world touchpoints where animal, human, and environmental health intersect.

**Table 1:** Questionnaire item characteristics, including question phrasing, response options, and format types.

Q#	Question	Answer (options)	Q-type
1	You prepare a fresh salad. Everything appears healthy–crisp, green, and clean. Yet, what if you are inadvertently becoming part of a global health challenge in the process? In your opin-ion, how likely is this? One answer possible.	1 = Very unlikely – that sounds like science fiction. 2 = Maybe there is a connection – but I consider it low. 3 = I am unsure – though it could be possible. 4 = That seems plausible to me – there is definitely more to it. 5 = Very likely – I suspect there is a real risk.	5-point Likert, SCQ
2	What could be a reason or reasons why consuming salad might pose a health risk that links animal husbandry AND the environment?	a) Residues of pesticides on the salad. b) **Bacteria from animal manure get onto the plants via fertiliser.** c) Packaging contains microplastics. d) Salad is washed with chlorinated water. e) Humans introduce germs to the food during transport.	MCQ
3	How urgent do you think it is that health problems are solved jointly across veterinary medicine, human medicine, and environmental sciences? One answer possible.	1 = Not urgent at all. 2 = Not very urgent. 3 = Moderately urgent. 4 = Quite urgent. 5 = Very urgent	5-point Likert, SCQ
4	Where do you encounter topics in your everyday life that affect animals, humans, and the environment together?	Open-ended free text.	OQ

SCQ, single choice question; MCQ, multiple choice question; OQ, open-ended question. Correct answer in bold.

The participants' demographics and the first three questions were analysed descriptively with Microsoft Excel (v.2408) and results were visualised with GraphPad Prism (v.11.0.2). The answers to the open-ended question (Q4) were deductively categorised into the following seven main categories with subcategories in brackets: Food & Consumption, Animal Contact & Husbandry (Farm animals, Companion animals), Environment & Pollution (Infrastructure), Health & Disease (Individual Health and Public Health), Everyday Life (Recreational Exposure, Professional Exposure), Agriculture, and Unclear/Missing (Supplementary Table 1). The prevalence of mentioned main categories and their combinations were visualised in an upset plot created with the R package UpSetR (v.1.4.0) [12].

## 3 Results and discussion

A total of 102 participants completed the questionnaire in paper format, while five participated online. The data confidentiality agreement was accepted by 92 participants; the remaining 15 respondents who did not provide consent were excluded from further analyses. Among the 92 included participants, 78.3% (*n* = 72) identified as female, 20.7% (*n* = 19) as male, and one participant identified as diverse. Of those reporting their age (*n* = 90), the mean age was 30.17 years (± SD = 14.9 years). The cohort showed a broad age distribution across predefined age categories (Fig. 1a). The largest group comprised participants aged 36–50 years (28.3%, *n* = 26), followed by those younger than 18 years (23.9%, *n* = 22). Participants older than 50 years represented the smallest age group (9.8%, *n* = 9), while two individuals chose not to disclose their age. Beyond age and gender, the study cohort displayed considerable heterogeneity regarding educational background and occupation (Supplementary Table 2).

**Fig. 1:**
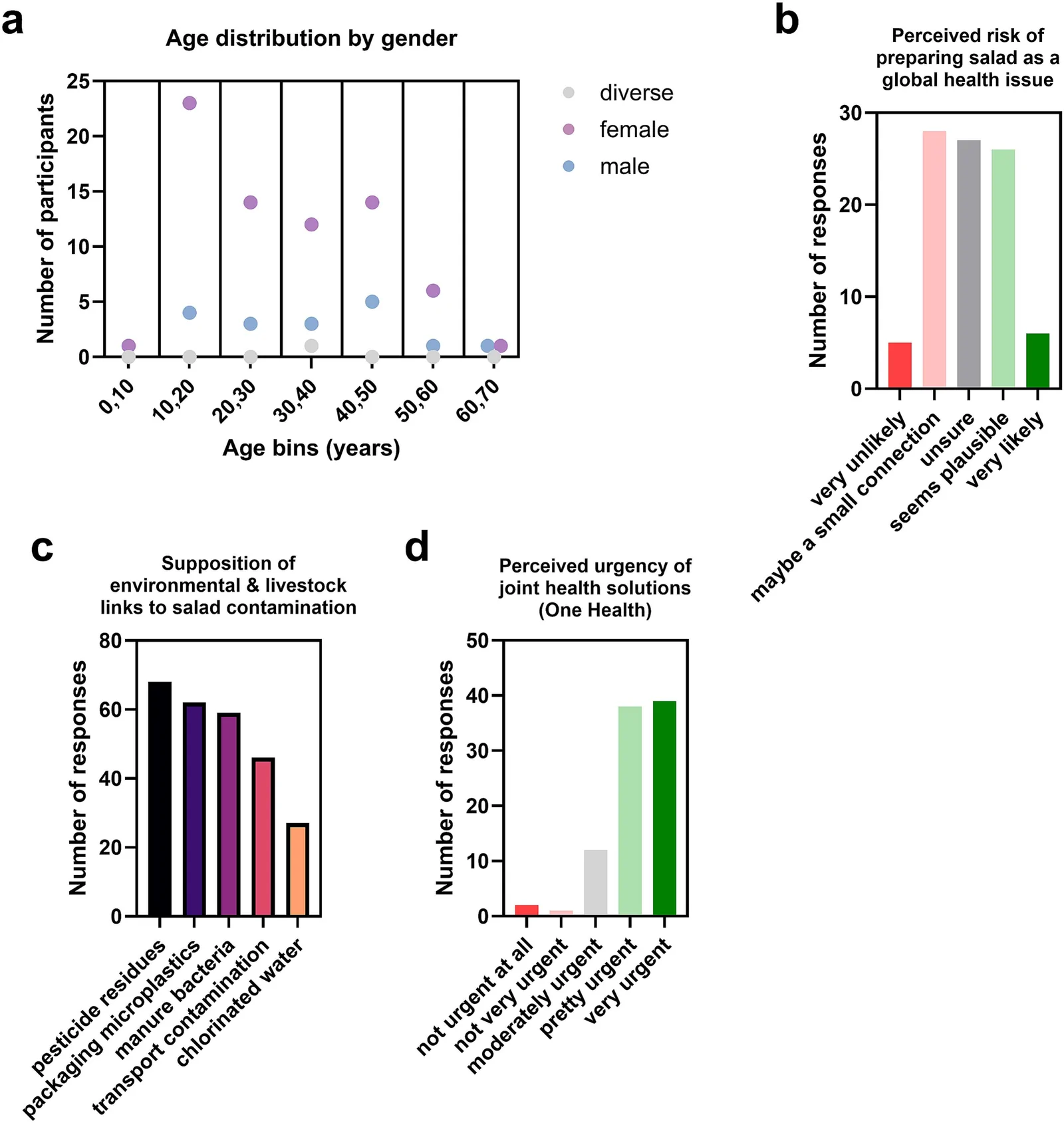
Demographic profile and public risk perception of “salad contamination” and OH solutions. (a) Age distribution of survey respondents grouped by gender identity across 10-year age bins. (b) Perceived risk connecting salad preparation to global health issues. (c) Perceived contribution of specific environmental and livestock factors to salad contamination ranked by absolute frequencies. (d) Perceived urgency of implementing joint, cross-disciplinary health solutions (OH framework).

### 3.1 Public perception and disciplinary interconnections of One Health

To evaluate how the public conceptualises OH interfaces in everyday life, participants were first presented with a routine food-preparation scenario. Regarding Q1, which assessed the perceived likelihood that preparing a fresh salad could be linked to a global health issue, most respondents demonstrated a cautious baseline awareness. Respondents clustered around intermediate risk perceptions, identifying the connection as “seems plausible” (28.3%, 26/92), “unsure” (29.3%, 27/92) or “maybe a small connection” (30.4%, 28/92) (Fig. 1b). In contrast, polar responses were rare; only 5.4% (5/92) evaluated the scenario as “highly unlikely”, and 6.5% (6/92) deemed it “very likely”. Overall, these findings suggest that many respondents intuitively recognised that everyday activities may be linked to broader health challenges, while lacking confidence regarding the underlying mechanisms. This observation is consistent with the concept of environmental health literacy. Gray (2018) noted that the general public frequently recognises environmental health risks in general terms but often struggles to identify specific exposure pathways or explain mechanisms connecting environmental factors and health outcomes. Thus, as seen in our sample, broad risk awareness does not necessarily reflect a detailed conceptual understanding of everyday health impacts [13].

To further assess technical knowledge regarding these mechanisms, Q2 prompted participants to identify specific pathways linking animal husbandry and the environment within the same “salad” scenario. Although posed as a single-choice question with a singular accurate pathway–*“Bacteria from animal manure get onto the plants via fertiliser”*–widespread multi-selection behaviour was observed, indicating a generalized rather than targeted risk perception. Cumulatively, environmental contaminants dominated lay concerns, with 62 respondents (67.4%) identifying microplastics as a primary health hazard in this context (Fig. 1c). When investigating granular choice patterns via an UpSet plot, the most prevalent response behaviour (20.7%, 16/92) was cross-selection of all available options (Supplementary Fig. 2). The second most frequent pattern combined microplastics and pesticides, whereas only 10.9% (10/92) correctly isolated the specific manure-fertiliser pathway. Rather than reflecting differentiated knowledge, this pattern suggests overgeneralisation as a response strategy, where participants attributed food-related risks to multiple environmental contaminants rather than identifying the specific transmission pathway in question. Similar response patterns have been reported in food safety research, where consumers frequently perceive a broad range of environmental and chemical hazards as important food-related risks, often placing greater emphasis on these than on microbiological contamination. Colacicco et al. (2023) argue that public risk perception is often driven by the prominence of widely discussed environmental issues rather than scientifically relevant transmission pathways [14]. Another study demonstrated that consumers often perceive multiple food-related hazards simultaneously, regardless of their actual relevance to a given exposure scenario [15]. Likewise, studies investigating the public understanding of foodborne pathogens have similarly identified knowledge gaps regarding foodborne diseases and microbial risks [16]. In line with this existing literature, our data confirm that lay risk assessments in this scenario were driven primarily by broad environmental anxieties rather than specific technical knowledge of core microbial transmission routes.

### 3.2 The paradox of perceived urgency versus fragmented literacy

Despite these knowledge gaps, participants expressed remarkably strong support for interdisciplinary health approaches. In Q3, respondents rated the urgency of addressing health challenges jointly across veterinary medicine, human medicine, and environmental sciences. The cohort demonstrated an overwhelming consensus: 83.7% (77/92) rated collaborative OH action as either “pretty urgent” or “very urgent” (Fig. 1d). Only a small fraction (3.3%, 3/92) deemed it of low priority. This strong directional consensus indicates high public motivation and a robust sociopolitical support for integrated health frameworks. Chiesa et al. (2021) similarly observed that while the value of interdisciplinary collaboration is widely acknowledged across Europe, only a minority can accurately define the OH concept, underscoring a disconnect between favourable attitudes and technical knowledge [17]. This discrepancy closely mirrors the pattern observed in our cohort, where participants strongly endorsed interdisciplinary collaboration despite demonstrating limited understanding of specific OH interfaces.

However, when stripped of the prompt-based framing of the “salad scenario” and asked in an open-ended format (Q4) to identify independent daily life spaces where animal, human, and environmental health intersect, this motivation failed to translate into comprehensive conceptual literacy. Spontaneous conceptualizations, visualised via an UpSet plot (Fig. 2), revealed that approximately one quarter (24/92, 26.1%) of respondents failed to provide scorable data, reinforcing lack of baseline confidence when being unguided by explicit contextual cues. Among scorable responses, isolated, single-category associations dominated over multidisciplinary or cross-linked reasoning. The data was heavily skewed toward the environmental domain, led by “Environment & Pollution” (*n* = 35), with which built infrastructure was explicitly cited in 20% (7/35) of cases. The second primary domain, “Everyday Life” (*n* = 28) remained bounded within routine human activities, balancing recreational (35.7%, 10/28) and professional (32.1%, 9/28) exposures. Crucially, the core animal and agricultural pillars defining the OH continuum were severely underrepresented. The category “Animal Contact & Husbandry” emerged in only 13 responses, with explicit companion animal or livestock interfaces restricted to just 15.4% (2/13) of those entries. Similarly, “Health & Disease” (*n* = 12) was conceptualized almost entirely as public health (41.7%, 5/12) rather than individual clinical health (8.3%, 1/12). Comparable patterns have recently been reported in surveys investigating OH awareness and knowledge. Wu et al. (2023) found that despite being unfamiliar with the formal term “One Health” the public recognises the general interconnectedness of human, animal, and environmental health [18]. Similarly, Amer et al. observed relatively high overall OH awareness among higher education students, but persistent gaps in specific OH elements including zoonotic diseases [19]. Likewise, our findings suggest that it is a challenge to translate implicit awareness into a comprehensive and spontaneously retrievable conceptual understanding.

**Fig. 2:**
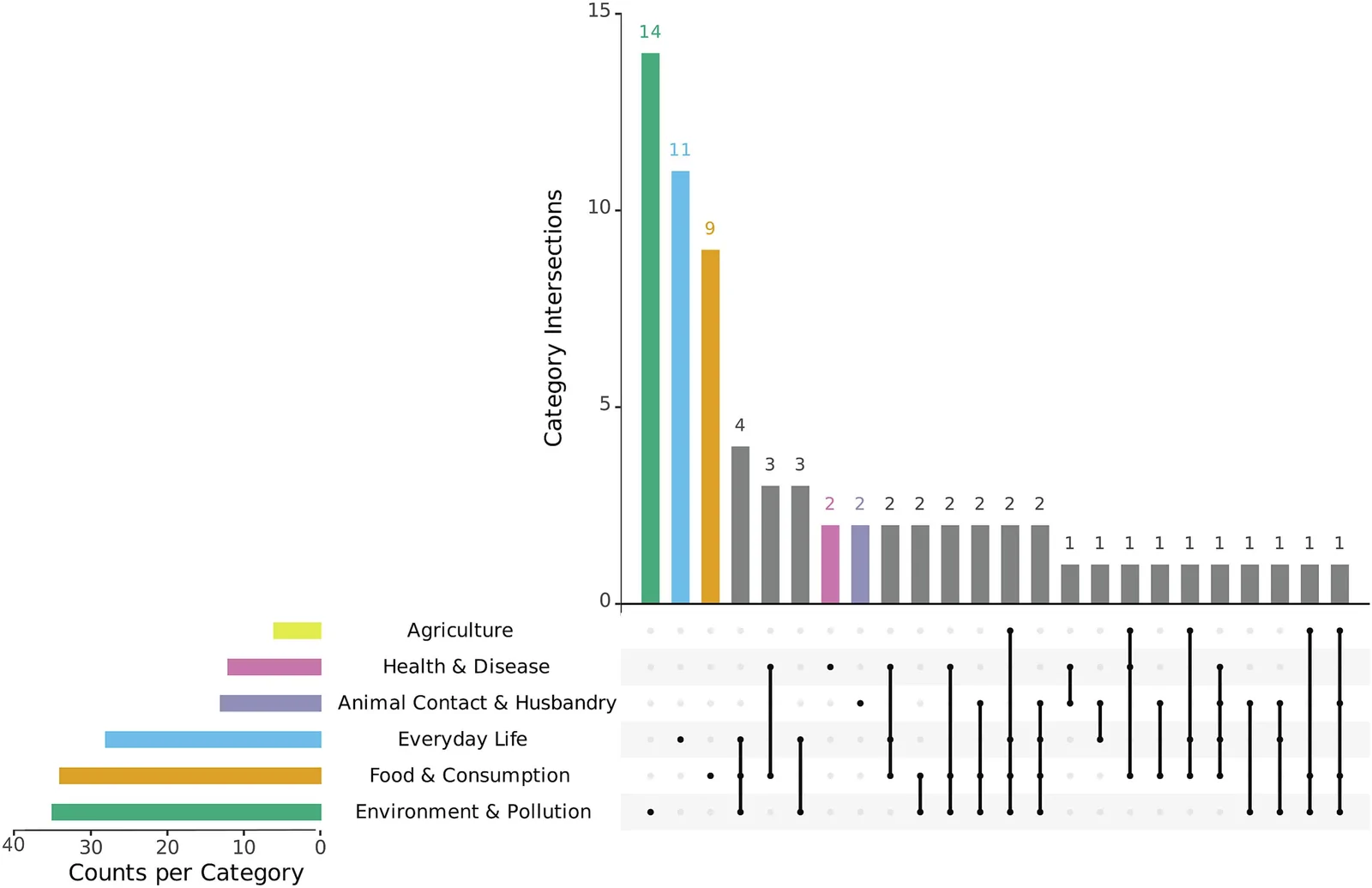
UpSet plot illustrating the response intersection pattern of defined categories. The horizontal bars on the left-hand side represent the number of times each category was given within the answers of the questionnaire in total (set size), while the vertical upper bars represent the times each combination of categories was given (intersection size). Colouring of upper bars represents single responses combinations.

Although the predominance of single-category assignments is partly influenced by the deductive categorization scheme, the overall pattern reflects a compartmentalised OH understanding. Public understanding remains focused on visible environmental and lifestyle factors, largely overlooking critical human-animal interfaces and interdisciplinary connections central to OH. Respondents appeared to conceptualise environmental issues, lifestyle, and health as largely separate domains rather than interconnected components of a complex system. This fragmented conceptualisation contrasts sharply with the strong support for interdisciplinary collaboration observed in Q3. Recent work has emphasized that successful OH implementation depends not only on public awareness but also on the development of OH literacy, defined as the knowledge, motivation, and competencies required to access, understand, evaluate, and apply OH-related information in everyday decision-making [20]. Our findings therefore suggest that future educational initiatives should move beyond mere awareness-raising and instead promote a holistic understanding of human, animal, and environmental health. The clear disparity between high moral motivation (Q3) and siloed baseline knowledge (Q4) underscores the urge for educational interventions capable of anchoring abstract systemic interdependencies within tangible, everyday frameworks.

This study has several limitations. First, recruiting participants at an OH info booth introduces inherent selection bias, and explicit references to animal husbandry and health risks in early questions may have primed subsequent open-ended responses. Second, the loud, busy open-house setting may have distracted respondents, reducing their motivation to spend time on the open-ended question. Third, the open-ended format may not have been fully age-appropriate for the younger children (aged <14 years) in the cohort. Finally, the modest sample size (*n* = 92) drawn from university visitors likely possesses a baseline interest in science and does not represent the broader Austrian public. Larger, more representative samples are required to adequately assess national OH perceptions.

## 4 Conclusions

In conclusion, however, our findings demonstrate a striking paradox in public risk perception: while systemic motivation and perceived urgency for OH initiatives are exceptionally high, baseline operational literacy remains highly fragmented, siloed, and disproportionately centred to environmental issues. This disconnect highlights a critical bottleneck for successful OH operationalisation. To effectively convert expressed societal urgency into accurate, actionable civic cooperation, there is an immediate need for targeted public education strategies that utilise brief, tangible, and routine lifestyle examples. This approach will directly align with the strategic mandates of the Quadripartite One Health Joint Plan of Action – headed by the FAO, UNEP, WOAH, and WHO – which prioritises public engagement and risk communication as indispensable tools for cultivating cross-sectoral understanding and systemic resilience [21].

## CRediT authorship contribution statement

**P. Saria:** Writing – original draft, Visualization, Project administration, Methodology, Formal analysis, Data curation, Conceptualization. **E.-M. Bartl:** Writing – review & editing, Writing – original draft, Methodology, Formal analysis, Data curation, Conceptualization. **S. Winkler:** Writing – review & editing, Writing – original draft, Methodology, Formal analysis, Data curation, Conceptualization. **R. Liermann:** Writing – review & editing, Methodology. **D. Ernst:** Writing – review & editing, Methodology. **S. Kau-Strebinger:** Writing – review & editing, Visualization, Supervision, Project administration, Methodology, Conceptualization.

## Ethics statement

This study involved human participants and was conducted in accordance with the principles of the Declaration of Helsinki. The Ethics Committee of the University of Applied Sciences Campus Vienna reviewed the project and granted a waiver of a formal ethics vote (EC No. 410/2026; application date: 01 July 2026), stating that it had no objections and that the project was in accordance with the Declaration of Helsinki. Participation was voluntary and anonymous. Participants were informed about the study purpose and survey requirements before participation, no personally identifiable data were collected, and submission of the anonymous questionnaire was considered informed consent. Appropriate procedures were used to protect participants' rights, privacy, and confidentiality.

## Ethics declaration

Written informed consent to take part in the study and to publish the article has been obtained from all participants or their legal representatives. The privacy rights of participants have been observed.

This study was performed in compliance with relevant laws, regulatory frameworks and guidelines where the research took place. The Ethics Committee of the University of Applied Sciences Campus Vienna granted an exemption. The authors provided the following explanation: The Ethics Committee of the University of Applied Sciences Campus Vienna has reviewed the submitted project, particularly regarding the principles of the Declaration of Helsinki, and hereby grants a waiver of a formal ethics vote for the submitted project. The main reasons for this are: The Ethics Committee has no objections to the application entitled “Perception of the One Health Concept among Visitors Attending the Open Day at the University of Veterinary Medicine Vienna: An Anonymous Cross-Sectional Survey”.

## Declaration of generative AI and AI-assisted technologies in the writing process

During the preparation of this work, the authors used Gemini 3.5 and 3.6 Flash (large language model, Google) and DeepL Translator (translation tool, DeepL SE) to improve language clarity, refine prose, and optimise the narrative flow. After using these tools, the authors reviewed, edited, and verified all outputs, and take full responsibility for the accuracy and integrity of the final manuscript.

## Funding

This project was funded by the One Health PhD Programme of the University of Veterinary Medicine Vienna, Austria.

## Declaration of competing interest

The authors declare that they have no known competing financial interests or personal relationships that could have appeared to influence the work reported in this paper.

## Data Availability

Aggregated data supporting the findings of this study are presented in the article. The survey was conducted anonymously, no personally identifiable data were collected, and individual-level responses are not reported. Additional information may be available from the corresponding author upon reasonable request, subject to institutional and ethical restrictions.
