# Supplementary material for: Urgent but isolated: Public perception and understanding of One Health in daily life
Source: One Health. 2026 Oct 16;23:101623. doi: 10.1016/j.onehlt.2026.101623 (PMC13720174; doi:10.1016/j.onehlt.2026.101623)
Supplement: Supplementary file 1 [file mmc1.pdf]

One Health Questionnaire – Your Opinion Counts!

We invite you to share your assessments on the topic of One Health with us through this short questionnaire. Thank you very much for your support!

About You

- **Age:** years
- **Gender:** ☐ female ☐ male ☐ diverse ☐ prefer not to say
- **Occupation / Training:** ____________________________

Privacy Policy:

The protection of your personal data is of particular importance to us in this survey. Your data is therefore collected and processed exclusively on the basis of the statutory provisions (§7 DSG, Art. 6() and Art. 89 GDPR, and FOG). This survey is conducted as part of a study at the University of Veterinary Medicine Vienna (Vetmeduni Vienna). The data may be accessed by the study team of the One Health PhD Programme of Vetmeduni. The data will not be passed on to third parties.
The collected data may be stored without restriction in accordance with Art. 89() GDPR. You have the right to obtain information from the controller of this study about the personal data collected, as well as the right to rectification, erasure, restriction of processing, and the right to object.
By withdrawing consent, the lawfulness of processing carried out on the basis of consent before its withdrawal remains unaffected. Please note, however, that personal data in the sense of research material pursuant to §2b(6) FOG may continue to be used.

If you have any questions about this survey, please contact the responsible person: Assoc. Prof. Dr. Silvio Kau-Strebinger (silvio.kau-strebinger@vetmeduni.ac.at), Center for Pathobiology, 1210 Vienna, Veterinärplatz 1.
For general legal questions regarding GDPR/DSG and FOG, please contact the data protection officer of Vetmeduni (datenschutz@vetmeduni.ac.at). You also have the right to lodge a complaint with the data protection authority (e.g., dsb@dsb.gv.at).

☐ **I agree to the privacy policy and to the scientific use of my data.**

Question **1**

You prepare a fresh salad. Everything appears healthy-crisp, green, and clean. Yet, what if you are inadvertently becoming part of a global health challenge in the process? In your opinion, how likely is this? One answer possible.

☐ 1 = Very unlikely – that sounds like science fiction. Maybe
☐ 2 = there is a connection – but I consider it low.
☐ 3 = I'm unsure – though it coule be possible.
☐ 4 = That seems plausible to me – there is definitely more to it.
☐ 5 = Very likely – I suspect there is a real risk.

One Health Questionnaire – Your Opinion Counts!

Question 2

What could be a reason or reasons why consuming salad might pose a health risk that links animal husbandry AND the environment?

☐ Residues of pesticides on the salad.
☐ Bacteria from animal manure get onto the plants via fertilizer.
☐ Packaging contains microplastics.
☐ Salad is washed with chlorinated water.
☐ People introduce germs onto the food during transport.

Question 3

How urgent do you think it is to solve health problems jointly across veterinary medicine, human medicine, and environmental sciences? One answer possible.

☐ 1 = not urgent at all
☐ 2 = not very urgent
☐ 3 = moderately urgent
☐ 4 = pretty urgent
☐ 5 = very urgent

Question 4

Where do you encounter topics in everyday life that concern animals, humans, and the environment together?

Free text / keywords

Many thanks for your participation and please continue eating lettuce! ☺

One Health Fragebogen – Ihre Meinung zählt!

Wir laden Sie mit diesem kurzen Fragebogen ein, Ihre Einschätzungen zum Thema One Health mit uns zu teilen. Herzlichen Dank für Ihre Unterstützung!

Über Sie

- Alter: ____ Jahre
- Geschlecht: ☐ weiblich ☐ männlich ☐ divers ☐ keine Angabe
- Beruf / Ausbildung: ______________________________

Datenschutzerklärung:

Der Schutz Ihrer persönlichen Daten ist uns bei dieser Befragung ein besonderes Anliegen. Ihre Daten werden daher ausschließlich auf Grundlage der gesetzlichen Bestimmungen (§7 DSG, Art. 6 (1) und Art 89 DSGVO und FOG) erhoben und verarbeitet. Diese Befragung wird im Rahmen einer Studie an der Veterinärmedizinischen Universität Wien erstellt. Die Daten können vom Studienteam des One Health PhD Programms der Vetmeduni eingesehen werden. Die Daten werden nicht an Dritte weitergegeben. Die erhobenen Daten dürfen gemäß Art 89 Abs 1 DSGVO unbeschränkt gespeichert werden. Es besteht das Recht auf Auskunft durch den/die Verantwortlichen an dieser Studie über die erhobenen personenbezogenen Daten sowie das Recht auf Berichtigung, Löschung, Einschränkung der Verarbeitung der Daten sowie ein Widerspruchsrecht. Durch den Widerruf der Einwilligung wird die Rechtmäßigkeit, der aufgrund der Einwilligung bis zum Widerruf erfolgten Verarbeitung der personenbezogenen Daten nicht berührt. Bitte beachten Sie jedoch, dass personenbezogene Daten im Sinne von Forschungsmaterial gemäß § 2b Abs 6 FOG weiterhin verwendet werden können!

Wenn Sie Fragen zu dieser Erhebung haben, wenden Sie sich bitte gern an den Verantwortlichen: Ass.-Prof. Dr. Silvio Kau-Strebinger (silvio.kau-strebinger@vetmeduni.ac.at), Zentrum für Pathobiologie, 1210 Wien, Veterinärplatz 1. Für grundsätzliche juristische Fragen im Zusammenhang mit der DSGVO/DSG und FOG wenden Sie sich an den/die Datenschutzbeauftragte:n der Vetmeduni (datenschutz@vetmeduni.ac.at). Zudem besteht das Recht der Beschwerde bei der Datenschutzbehörde (bspw. über dsb@dsb.gv.at).

☐ **Ich stimme der Datenschutzerklärung und wissenschaftlichen Verwendung meiner Daten zu.**

Frage 1

Sie bereiten einen frischen Salat zu. Alles sieht gesund aus – knackig, grün, sauber. Doch was, wenn Sie dabei unbeabsichtigt Teil eines weltweiten Gesundheitsproblems werden? Wie wahrscheinlich ist das Ihrer Meinung nach? Eine Antwort möglich.

☐ 1 = Sehr unwahrscheinlich – das klingt nach Science-Fiction.
☐ 2 = Vielleicht gibt es einen Zusammenhang – den halte ich aber für gering.
☐ 3 = Ich bin unsicher – möglich wäre es schon.
☐ 4 = Das erscheint mir plausibel – da steckt bestimmt mehr dahinter.
☐ 5 = Sehr wahrscheinlich – ich vermute, dass da ein echtes Risiko besteht.

One Health Fragebogen – Ihre Meinung zählt!

Frage 2

Was könnte ein Grund / könnten Gründe sein, warum beim Salatessen ein Gesundheitsrisiko entstehen kann, das mit Tierhaltung UND Umwelt zusammenhängt?

☐ Rückstände von Pflanzenschutzmitteln auf dem Salat.
☐ Bakterien aus Tierdung gelangen über Dünger auf die Pflanzen.
☐ Verpackungen enthalten Mikroplastik.
☐ Salat wird mit chlorhaltigem Wasser gewaschen.
☐ Menschen bringen Keime beim Transport auf die Lebensmittel.

Frage 3

Wie dringend finden Sie es, dass Gesundheitsprobleme gemeinsam über Tiermedizin, Humanmedizin und Umweltwissenschaften hinweg gelöst werden? Eine Antwort möglich.

☐ 1= überhaupt nicht dringend
☐ 2 = wenig dringend
☐ 3 = mittelmäßig dringend
☐ 4 = ziemlich dringend
☐ 5 = sehr dringend

Frage 4

Wo begegnen Ihnen im Alltag Themen, die Tier, Mensch und Umwelt gemeinsam betreffen?

Freitext / Stichwörter

Herzlichen Dank für Ihre Teilnahme und bitte essen Sie weiterhin Salat! ☺
