# Supplementary material for: Urgent but isolated: Public perception and understanding of One Health in daily life
Source: One Health. 2026 Oct 16;23:101623. doi: 10.1016/j.onehlt.2026.101623 (PMC13720174; doi:10.1016/j.onehlt.2026.101623)
Supplement: Supplementary file 2 [file mmc2.pdf]

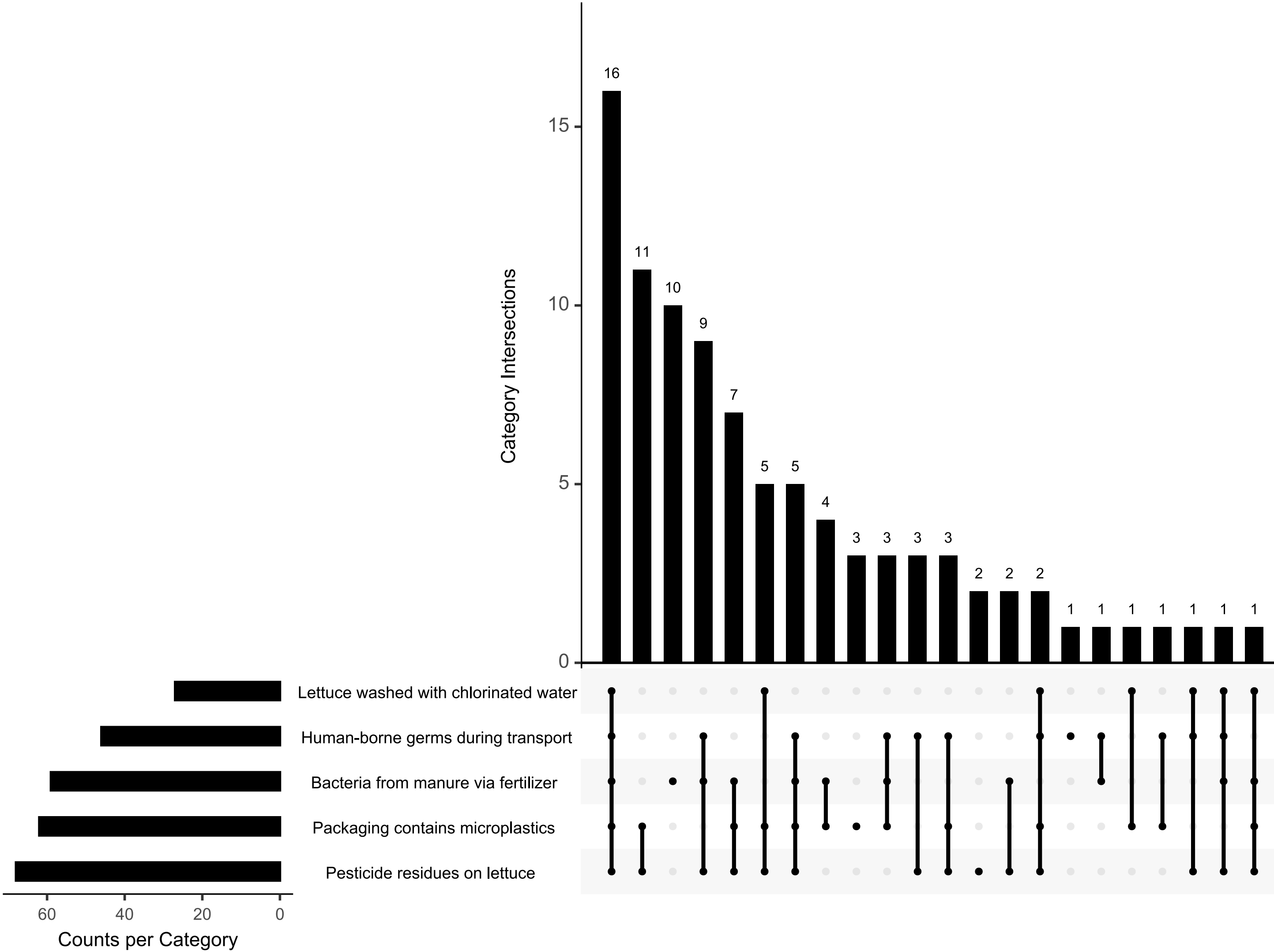

Category Intersections
16
11
10
9
7
5
5
4
3
3
3
3
2
2
2
1
1
1
1
1
1
1
0
5
10
15
Lettuce washed with chlorinated water
Human-borne germs during transport
Bacteria from manure via fertilizer
Packaging contains microplastics
Pesticide residues on lettuce
60
40
20
0
Counts per Category
